# Indocyanine Green as a Marker for Tissue Ischemia in Spinal Tumor Resections and Extended Revisions: A Technical Note

**DOI:** 10.3390/jcm14030914

**Published:** 2025-01-30

**Authors:** Max Ward, Daniel Schneider, Ethan D. L. Brown, Apratim Maity, Barnabas Obeng-Gyasi, Roee Ber, Aladine A. Elsamadicy, Daniel M. Sciubba, Denis Knobel, Sheng-Fu Larry Lo

**Affiliations:** 1 Department of Neurological Surgery, Donald and Barbara Zucker School of Medicine at Hofstra/Northwell, Lake Success, NY 11549, USA; mward7@northwell.edu (M.W.); dschneider5@northwell.edu (D.S.); ebrown35@northwell.edu (E.D.L.B.); amaity@northwell.edu (A.M.); berlerner@gmail.com (R.B.); dsciubba1@northwell.edu (D.M.S.); 2 Department of Neurological Surgery, Indiana University School of Medicine, Indianapolis, IN 46202, USA; bobenggy@iu.edu; 3 Department of Neurological Surgery, Yale School of Medicine, New Haven, CT 06510, USA; aladine.elsamadicy@yale.edu; 4 Department of Plastic Surgery, Donald and Barbara Zucker School of Medicine at Hofstra/Northwell, Lake Success, NY 11549, USA; dknobel@northwell.edu

**Keywords:** indocyanine green (ICG), case series, perfusion, spine, spinal oncology, en-bloc resection

## Abstract

**Background/Objectives:** The increasing complexity of spinal oncology procedures, particularly in en-bloc tumor resections, creates challenges in tissue perfusion assessment due to extended operative times and extensive surgical dissection. Real-time visualization of tissue perfusion can be achieved with ICG using commercially available handheld imaging systems, offering potential advantages in spinal oncology cases. This study assessed the utility of ICG in analyzing soft-tissue viability during complex spine procedures extending beyond 7.5 h, with a particular focus on oncologic resections. **Methods**: Three cases that required over 7.5 h of operative time were chosen for ICG utilization. These cases included an en-bloc malignant peripheral nerve sheath tumor resection, an en-bloc resection of a malignant epithelioid neoplasm, and a long-segment fusion revision for pseudoarthrosis. At the conclusion of the critical portion of the procedure, a handheld intraoperative fluorescence camera was utilized to visualize the tissue penetration of intravenous ICG. **Results**: Prior to injecting ICG, devascularized tissue was not clearly visible. Injecting ICG allowed clear separation of vascularized (fluorescing) and devascularized (non-fluorescing) tissues. One region of non-florescent tissue was later confirmed to be devascularized with MRI and experienced postoperative infection. **Conclusions**: As the complexity of spinal oncology procedures increases, ICG fluorescence imaging offers a novel method for real-time assessment of tissue perfusion. This technique may be particularly valuable in extensive tumor resections, post-radiation cases, and revision surgeries where tissue viability is at risk. Further investigation in the spinal oncology population could help establish whether early identification of poorly perfused tissues impacts wound healing outcomes.

## 1. Introduction

As the aging population continues to grow, the incidence of spinal oncologic pathologies has increased correspondingly [1], necessitating complex surgical interventions with extended operative times. En-bloc spinal tumor resection, particularly in cases of primary malignancies, often requires extended periods of tissue retraction, larger exposures, and management of significant blood loss [2]. These technical demands are further complicated in patients who have undergone prior radiation therapy, where tissue planes may be distorted and vascularity may be compromised [3]. In these challenging cases, the spinal musculature is exposed to various forms of hemodynamic compromise and may be prone to intraoperative ischemia and death. This tissue may ultimately complete its infarction intraoperatively without obvious changes on visual inspection. Postoperatively, such dead or necrotic tissue may serve as a nidus for infection. Given the increased rates of infection in spinal surgeries with extended operative times, assessing tissue ischemia at the conclusion of surgery could serve as an important step in reducing this risk [4,5]. Despite this, as of yet there is no published protocol for evaluating tissue viability at the end of complex spinal tumor surgery or even spinal surgery in general.

Although visual inspection can be reliable for dermal tissue, muscle has various blood supplies and thus may not appear visually as non-viable on initial gross examination. Indocyanine green (ICG) dye, a compound that has been effectively utilized since the 1950s, might help address existing deficiencies in the assessment of tissue viability [6]. ICG emits light within the infrared spectrum, and it more easily penetrates deeper tissues for visualization compared to other dyes. This compound is safe, with few potential side effects, and has been utilized in noninvasive angiography, tumor and lymph node identification, and more recently tissue perfusion studies [7,8,9]. The application of ICG in spinal oncology surgery represents a potentially valuable tool for optimizing surgical outcomes, particularly in cases requiring extensive dissection or in patients with compromised tissue quality due to prior treatments.

The SPY-Portable Handheld Imager (PHI) camera system can acquire images with a 1080p resolution at 60 frames per second. This system combines fluorescence signal information with vivid white-light imaging in real time, allowing detailed visualization of tissue perfusion. The camera provides multiple visualization modes, including white-light, SPY Fluorescence, Overlay, and Color-Segmented Fluorescence (CSF) modes. This study aimed to assess the utility of ICG in analyzing soft-tissue viability by incorporating the SPY-PHI camera system into the postoperative visualization of three complex spine cases that extended over 7.5 h, paying particular attention to cases involving oncologic pathologies.

## 2. Materials and Methods

This study did not require approval by the Institutional Review Board (IRB), as it included three or fewer patients. Consent was not obtained, as ICG is routinely used during plastic surgery as a standard tool to assess tissue perfusion and the results were deidentified. While its use in spine surgery is uncommon, it is on-label for the general assessment of tissue perfusion within surgical procedures and does not require IRB approval prior to use. Three non-consecutive cases that required over 7.5 h of operative time were chosen for ICG utilization. These cases were treated at a single academic medical institution. These cases included one en-bloc malignant peripheral nerve sheath tumor resection, one separation surgery for a malignant epithelioid neoplasm, and one long-segment fusion revision for pseudoarthrosis. A retrospective review of the patients’ charts was conducted to collect data on operative details, postoperative outcomes, and the occurrence of any re-operations.

After completely excising the relevant pathologic tissue, the Stryker SPY-PHI camera (Stryker Corporation, Kalamazoo, MI, USA) was sterilely draped and brought onto the field. This camera was chosen over a more generally available surgical microscope due to its lower profile, ease of draping for surgery, and variety of modes for quantifying perfusion. Retractors were removed from the operative field. Next, 25 mg of ICG powder was sterilely reconstituted in 10 cc of sterile saline, and 3 cc of dye (12.5 mg of ICG) was injected intravenously. The camera was used to visualize the tissue perfusion within the postoperative bed while employing the black–white filter mode. ICG fluorescence was qualitatively assessed by the neurosurgeons and plastic surgeons involved in the cases, and images were recorded. All surgeries were performed by the same neurosurgery and plastic surgery teams. This case series has been reported according to the PROCESS Guideline [10].

In order to elucidate any possible previous uses of this technique in spine surgery, a comprehensive literature review was conducted with the assistance of a medical librarian. The utilized search terms are contained within Supplementary Materials S1.

A supplementary literature review was subsequently conducted with the assistance of a medical librarian to investigate any previous uses of ICG fluorescence in spine surgery (Supplementary Materials S1). All articles published between 12 January 2015 and 12 January 2025 were considered for inclusion in this article. Articles related to ICG fluorescence utilized for the assessment of muscle perfusion in the course of a spinal surgery were included, while non-English works were excluded. Ultimately, 770 articles indexed on PubMed between 12 January 2015 and 12 January 2025 referencing terms such as “Indocyanine green”, “surgery”, “spine”, and “tissue perfusion” were reviewed for analysis.

## 3. Results

A complete summary of the cases is presented in Table 1.

### 3.1. Case 1

**Figure 1:**
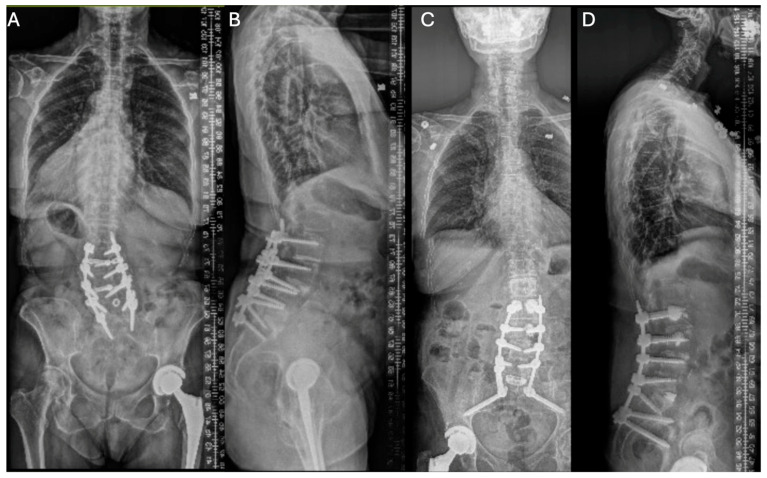
Case 1: pre- versus postoperative standing X-rays. Preoperative AP (**A**) and lateral (**B**) standing scoliosis X-rays showing a coronal deformity, a mild sagittal imbalance, and a loss of lumbar lordosis. Postoperative AP (**C**) and lateral (**D**) images showing an improved coronal deformity, lumbar lordosis, and a sagittal imbalance.

**Figure 2:**
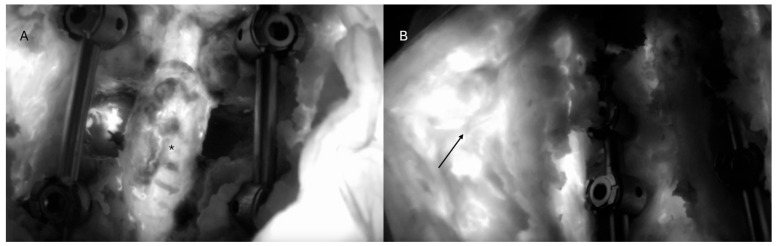
ICG perfusion of the thecal sac and deep muscles. (**A**) Post-ICG injection image showing good fluorescence of the thecal sac (*). (**B**) Post-injection image showing fluorescence of the muscle flap (arrow) with minimal perfusion deficits.

**Figure 3:**
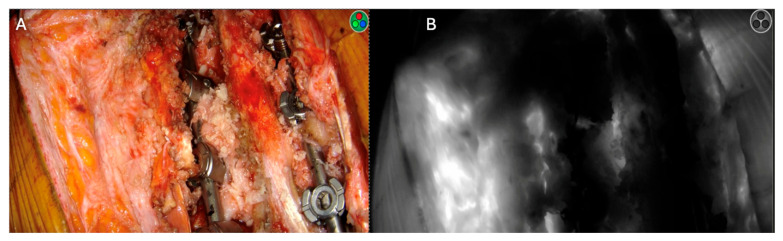
Intraoperative images showing (**A**) white-light and (**B**) ICG views of the operative field. There are no clear differences in tissue appearance in the white-light image. However, under ICG fluorescence, there were areas of tissue that did not fluoresce.

A 71-year-old female with a history of prior L2 to S1 spinal fusion presented with mechanical back pain and a sagittal plane deformity from proximal junctional kyphosis, as well as preoperative imaging suggestive of pseudoarthrosis (Figure 1). Revision surgery involved posterior L2 to pelvis exposure, examination of the previous fusion with confirmation of pseudoarthrosis, removal and upsizing of the spinal instrumentation, decompression, osteotomies, and arthrodesis. Intraoperative evaluation confirmed degenerative changes with pseudoarthrosis, and she was discharged 17 days later. ICG showed minimal tissue ischemia intraoperatively and good perfusion of the deep muscles and thecal sac (Figure 2). Notably, white light showed no clear visual differences between the florescent and non-florescent tissues, unlike ICG (Figure 3). She was last seen for a 10-month follow-up and showed no signs of incisional or deep wound complications.

### 3.2. Case 2

**Figure 4:**
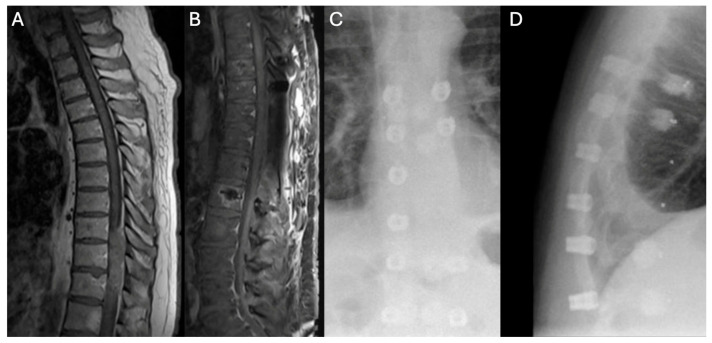
Case 2: pre- versus postoperative MRI. (**A**) Preoperative T1 post-contrast MRI showing enhanced T9–T12 extradural mass. (**B**) Postoperative T1 fat-sat post-contrast MRI showing removal of the enhanced tumor. (**C**) AP and (**D**) lateral chest X-rays showing the placement of the carbon-fiber pedicle screws and rods.

**Figure 5:**
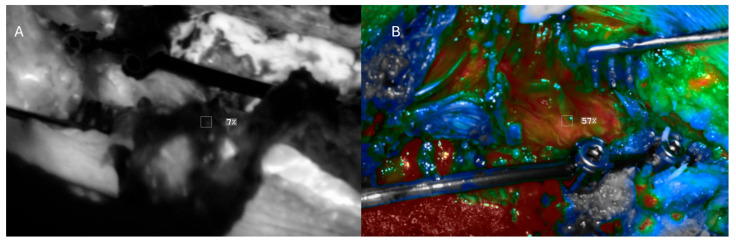
Deep-muscle perfusion with peripheral ischemia. (**A**) Black and white ICG imaging showing small areas of perfusion defects, as indicated by the ROI box showing 7% relative fluorescence. (**B**) Color-scaled imaging showing good perfusion of the underlying muscle flaps (white ROI box showing 57% fluorescence), indicated in red, with some areas of deficit indicated in blue.

A 59-year-old male presented with a five-day history of low back pain and right lower-extremity weakness and was diagnosed with a complex spinal canal mass spanning from T9 to T12 causing high-grade cord compression. He underwent complete resection of the tumor, decompression, facetectomies, pediculotomies, and instrumented fusion (Figure 4). Pathology revealed a malignant epithelioid neoplasm with INI1/SMARCB1 deficiency. Postoperatively, the patient underwent both chemotherapy and radiation therapy. No postoperative infections were observed. ICG showed good deep-muscle perfusion with tissue ischemia only along the periphery of the wound (Figure 5).

### 3.3. Case 3

**Figure 6:**
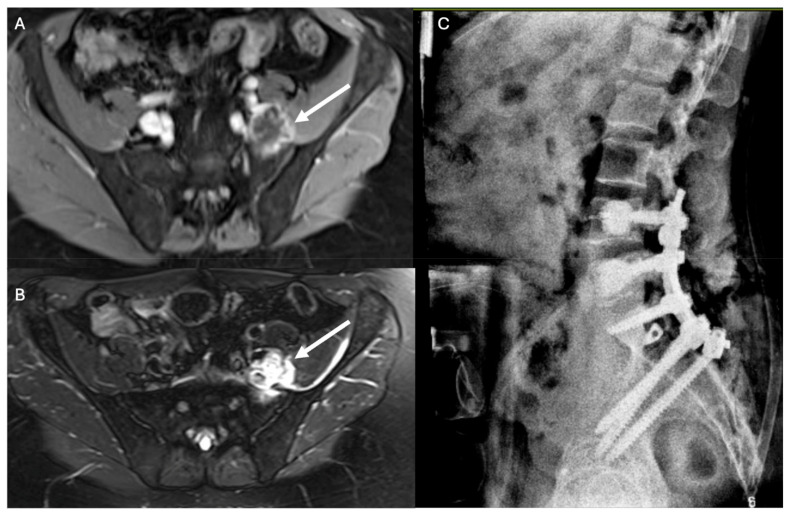
Case 3: pre- versus postoperative imaging. Preoperative T1 post-contrast (**A**) and T2 (**B**) MRI showing the left-sided L4 nerve sheath tumor as indicated by white arrow. (**C**) Postoperative X-ray showing L4–pelvis instrumentation after nerve sheath tumor removal.

**Figure 7:**
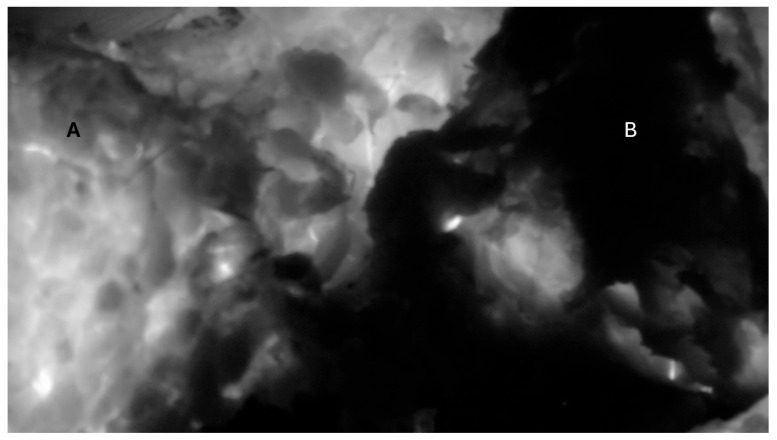
The non-fluorescence of the gluteal muscle flap. Post-ICG injection black–white imaging showing two segments of the gluteal flap. Segment (**A**) showed good fluorescence, white segment (**B**) showed no fluorescence after the supplying artery was coagulated.

**Figure 8:**
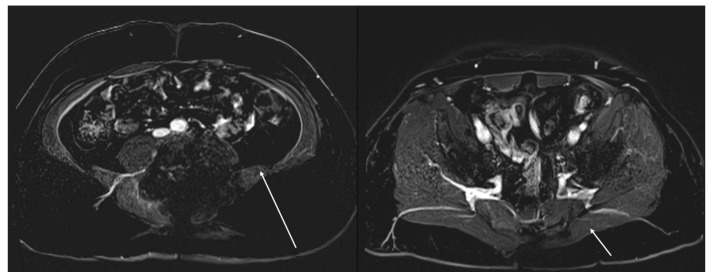
Postoperative MRI indicating asymmetric tissue perfusion. Postoperative MRI scans demonstrating asymmetry in contrast filling (white arrows), suggestive of a possible mismatch in tissue perfusion. The left image reveals reduced perfusion in the postoperative bed, while the right image shows more uniform perfusion. The asymmetry indicated by the arrows may reflect tissue ischemia or a compromised postoperative blood supply.

A 44-year-old male with a history that included a non-seminomatous germ-cell tumor that was previously treated with chemotherapy and radiation presented with an enlarging L4 nerve sheath tumor causing significant quadriceps weakness and sacral erosion. The patient underwent tumor resection, high hemi-sacrectomy, iliac osteotomy, and instrumented fusion (Figure 6). Pathology confirmed a high-grade spindle-cell sarcoma consistent with a malignant peripheral nerve sheath tumor (MPNST). ICG intraoperatively showed a completely non-fluorescent flap of gluteal muscle after the required lateral dissection interrupted the arterial blood supply (Figure 7). The tissue was not debrided at that time, as the plastic surgeon felt the defect may have been too extensive to close primarily without significant tissue flapping. A postoperative MRI showed a potential perfusion defect in the muscle (Figure 8). One month postoperatively, the patient developed a wound infection, which was managed with surgical debridement. The patient then underwent seven weeks of proton beam therapy. The infection recurred seven months later and was again debrided and treated with antibiotics.

## 4. Discussion

The successful management of spinal oncology cases increasingly requires sophisticated approaches to tissue handling and perfusion monitoring, particularly in settings with prior radiation therapy or extensive surgical dissection. Our findings suggest that ICG-based perfusion assessment presents a promising tool for identifying compromised tissue during these complex procedures. This technique may be particularly valuable in cases requiring extensive dissection for en-bloc resection or in patients with tissue compromised by prior treatments. Visualization of tissue perfusion during spinal surgery is a promising area of research due to its possible utility in the early identification of tissue ischemia. Despite these benefits, commonly employed techniques remain limited. This study suggests ICG dye as a possible modality for use due to its extensive history of safety, ease of use, and potential to translate to postoperative outcomes [11,12].

Notably, the use of ICG to identify and define the extent of lesions has a long history in spine surgery. Raabe et al. were the first to comment on its potential utility in the visualization of spinal perfusion in 2003, when they described the post-treatment occlusion of a spinous dural fistula [13]. This approach has been replicated across many settings, including the localization of hemangioblastomas, intramedullary tumors, peripheral nerve tumors, and arteriovenous malformations [14,15,16,17,18,19,20,21,22,23].

Despite this interest, studies investigating ICG in the perfusion assessment of non-pathologic spinal tissue or musculature have been more limited [24]. Imaging of the spinal cord microvasculature with ICG has been an area of limited interest, with several authors proposing it as a method to avoid injury to viable neural tissue during microsurgical interventions such as myelomeningocele repair [25,26,27]. Koyama et al., in a 2022 case report, described the isolated use of ICG to detect necrotic tissue margins in a 75-year-old woman suffering from a pelvic defect after several sacral debridements were required to manage tissue necrosis in the aftermath of sacral chordoma treatment [28]. Similarly, Acerbi et al., in a retrospective analysis of 93 instances of ICG FLOW 800 use in cranial and spinal tumors, identified a subset of cases involving post-resection applications in spinal tumors [29]. Although the post-resection use of ICG in spinal lesions was limited to only six cases, the authors reported two cranial cases in which hypoperfusion led to additional resections. Additionally, one instance of hypoperfusion in the optic chiasm led to permanent vision loss, despite the normal appearance of the affected region under white-light microscopy.

These findings have direct implications for spinal oncology surgery, where tissue perfusion faces multiple compromising factors. Radiation therapy can induce obliterative endarteritis and tissue fibrosis, reducing the baseline vascularity of surgical planes [30]. Studies have demonstrated that therapeutic radiation doses can significantly impair tissue oxygenation and reduce capillary density in muscle tissue [31,32]. In revision surgeries following radiation, the combination of treatment-induced fibrosis and postsurgical scarring creates particularly challenging conditions for wound healing. Previous findings have shown higher wound complication rates in preoperatively irradiated spine cases compared to non-irradiated cases [33]. However, as radiation techniques have evolved, more recent data have challenged this historical concern. Vargas et al. demonstrated that there were no significant differences in wound complication rates between patients receiving preoperative radiation (14.3%), postoperative radiation (10.8%), or no radiation (11.5%) in a contemporary cohort of over 200 metastatic spine patients [34]. This shift may reflect improvements in radiation delivery precision and surgical techniques, suggesting that historical concerns about preoperative radiation may need to be reconsidered going forward in the modern treatment era.

Recent studies have further validated the utility of ICG in both surgical margin assessment and wound repair applications [35,36]. Huang et al. demonstrated in a prospective trial of 70 bone and soft-tissue tumor patients that NIR imaging with ICG achieved positive margins in only 2/55 cases (3.6%), with particularly strong results in sarcoma cases (1/40, 2.5%) [35]. Their findings showed that ICG fluorescence was especially effective for tumors ≥5 cm in size and primary malignancies compared to benign or metastatic lesions, suggesting optimal scenarios for ICG implementation. Additionally, Miao et al. recently reported on a novel nanoplatform combining ICG with magnesium-incorporated mesoporous bioactive glass and a polydopamine coating that achieved both enhanced photodynamic therapy through ROS generation and low-temperature photothermal therapy around 45 °C [36]. This dual mechanism not only provided effective antibacterial activity but also promoted accelerated wound healing through immunomodulation of macrophage polarization. Their findings align with our observations regarding the benefits of moderate-temperature PTT while highlighting potential optimization strategies using nanoparticle delivery systems. The combination of precise margin assessment capabilities and wound healing promotion through carefully controlled photothermal effects underscores ICG’s versatility in surgical applications. As demonstrated in both recent studies and our current work, the ability to achieve these beneficial effects while maintaining temperatures below tissue-damaging thresholds represents a significant advantage [35,36].

ICG perfusion is more commonly utilized in other surgical fields. Surgeons specialized in skull base reconstruction have investigated intraoperative ICG fluorescence for over a decade as a predictor of MRI enhancement, flap necrosis, flap perfusion, infection, and CSF leaks [37,38,39,40]. While the rare nature of these postoperative complications precluded these studies from showing statistical relationships, Shaikh et al., in a recent systematic review of these cases, identified a strong association between ICG enhancement and postoperative MRI enhancement [41]. Similarly, Hitier et al., in a prospective study of twenty patients, identified vascular complications in all three patients that did not demonstrate flap fluorescence intraoperatively [42]. In related reconstructive surgeries including breast, abdominal wall, and peripheral tissue, ICG non-enhancement of flaps has been associated with reduced wound healing, increased postoperative complications, and severe flap necrosis [43,44,45,46]. In complex abdominal wall reconstruction, perhaps the closest parallel to complex spinal surgery currently studied, Wormer et al. identified a strong association between flap hypoperfusion and postoperative infection in a double-blind randomized controlled trial of 95 patients [12]. Despite this, intraoperative adjustment of hypoperfused flaps failed to significantly improve the complication rate in their study, although other studies have reported conflicting results [45].

Regardless, existing strategies for the use of intraoperative ICG to assess tissue perfusion can be easily applied in complex spinal surgery. The cost-effective nature of ICG in the setting of breast reconstruction has been previously described, with a per-vial cost of approximately USD 225, and this cheap and technically simple method could be used to reduce complication rates [37,47,48]. This is particularly true given the high baseline complication rate of flaps in spinal oncology patients, who face a 7.4% rate of postoperative wound infection [5,49]. Moreover, efficacious methods have already been described by Colavita et al. in complex abdominal surgeries, where an absolute perfusion unit threshold of ten and a 5 mg dose of ICG using the SPY Elite^®^ system (Stryker Corporation, Kalamazoo, MI, USA) produced 100% sensitivity and a specificity of 91% [50]. Consequently, future research into the predictive and clinical value of ICG perfusion studies following complex spinal surgery holds great potential to meaningfully alter existing clinical practice.

### Limitations

Despite our findings, this study possessed several limitations. Firstly, this was a small retrospective case series of only three patients, which limits the generalizability of our observations. Our findings of indocyanine green non-fluorescence prior to postoperative spinal surgery flap infection remain anecdotal and require further validation in larger cohorts. Prospective studies with larger sample sizes and controlled variables are needed to confirm this technique’s utility.

## 5. Conclusions

ICG and intraoperative utilization of a handheld fluorescence camera represent a viable method for assessing tissue perfusion in spinal oncology procedures and complex revisions. While our findings demonstrate that ICG provides safe and effective assessments of muscle perfusion, which are particularly valuable in extended tumor resections and post-radiation cases, the impact of intraoperative muscle ischemia on wound healing and infection rates in the oncologic population requires further investigation.

## Figures and Tables

**Table 1:** Summary of patient demographics, surgical details, and ICG findings ^1^.

Case	Age	Gender	Medical History	Pathology	Levels Exposed	Operative Time (h)	EBL (mL)	Postoperative Infection	Postoperative Radiation	Postoperative Chemotherapy	Length of Hospital Stay (Days)	Follow-Up Duration (Months)	ICG Findings
1	71	Female	Arthritis, HTN	Degenerative/pseudoarthrosis	L2–pelvis	7.5	500	No	No	No	17	10	Good tissue perfusion
2	59	Male	No major medical history	Malignant epithelioid neoplasm with INI1/SMARCB1 deficiency	T8–L1	10.5	1000	No	Yes	Yes	14	11	Muscle-edge tissue ischemia only
3	44	Male	Non-seminomatous germ-cell tumor treated with prior chemotherapy and radiation	High-grade spindle-cell sarcoma (MPNST)	L3–pelvis	12.25	500	Yes	Yes	No	7	10	Gluteal flap ischemia

This table outlines patient demographics, medical history, pathology, surgical details (levels exposed, operative time, and estimated blood loss [EBL]), postoperative complications, length of hospital stay, and ICG perfusion findings. Abbreviations: EBL, estimated blood loss; ICG, indocyanine green; MPNST, malignant peripheral nerve sheath tumor.

## Data Availability

The data utilized in this study are not available due to patient privacy restrictions.

## Supplementary Materials

The following supporting information can be downloaded at: https://www.mdpi.com/article/10.3390/jcm14030914/s1, the search terms utilized for the accompanying literature review.

## Abbreviations

IRB	Institutional Review Board
ICG	indocyanine green
PHI	Portable Handheld Imager
MRI	Magnetic Resonance Imaging
EBL	estimated blood loss
MPNST	malignant peripheral nerve sheath tumor
ROI	Region of Interest
