# Supplementary material for: Indocyanine Green as a Marker for Tissue Ischemia in Spinal Tumor Resections and Extended Revisions: A Technical Note
Source: J Clin Med. 2025 Jan 30;14(3):914. doi: 10.3390/jcm14030914 (PMC11818688; doi:10.3390/jcm14030914)
Supplement: Supplementary file 1 [file jcm-14-00914-s001.zip › jcm-3430106-supplementary.pdf]

Supplemental 1. Search Terms

PubMed

Limited to English articles published 01/12/15-01/12/25

Search 1

- ("Indocyanine green"[All Fields] AND ("spine"[MeSH Terms] OR "spine"[All Fields] OR "spines"[All Fields] OR "spine s"[All Fields] OR ("spinal"[All Fields] OR "spinalization"[All Fields] OR "spinalized"[All Fields] OR "spinally"[All Fields] OR "spinals"[All Fields]) OR "neuro*"[All Fields]) AND ("surgery"[MeSH Subheading] OR "surgery"[All Fields] OR "surgical procedures, operative"[MeSH Terms] OR ("surgical"[All Fields] AND "procedures"[All Fields] AND "operative"[All Fields]) OR "operative surgical procedures"[All Fields] OR "general surgery"[MeSH Terms] OR ("general"[All Fields] AND "surgery"[All Fields]) OR "general surgery"[All Fields] OR "surgery s"[All Fields] OR "surgerys"[All Fields] OR "surgeries"[All Fields])) AND ((y_10[Filter]) AND (english[Filter]))

Search 2

- "Indocyanine green"[All Fields] AND ((("tissue s"[All Fields] OR "tissues"[MeSH Terms] OR "tissues"[All Fields] OR "tissue"[All Fields]) AND ("perfusable"[All Fields] OR "perfusate"[All Fields] OR "perfusates"[All Fields] OR "perfuse"[All Fields] OR "perfused"[All Fields] OR "perfuses"[All Fields] OR "perfusing"[All Fields] OR "perfusion"[MeSH Terms] OR "perfusion"[All Fields] OR "perfusions"[All Fields])) OR ("muscle s"[All Fields] OR "muscles"[MeSH Terms] OR "muscles"[All Fields] OR "muscle"[All Fields] OR ("ischaemia"[All Fields] OR "ischemia"[MeSH Terms] OR "ischemia"[All Fields] OR "ischaemias"[All Fields] OR "ischemias"[All Fields]))) AND ("spine"[MeSH Terms] OR "spine"[All Fields] OR "spines"[All Fields] OR "spine s"[All Fields] OR ("spinal"[All Fields] OR "spinalization"[All Fields] OR "spinalized"[All Fields] OR "spinally"[All Fields] OR "spinals"[All Fields])) AND ((y_10[Filter]) AND "english"[Language])
